# The insufficient publication of early-phase clinical trials in oncology

**DOI:** 10.1093/oncolo/oyag361

**Published:** 2026-09-12

**Authors:** Merel J J Lucassen, Tim Schutte, Alwin D R Huitema, Neeltje Steeghs

**Affiliations:** Department of Medical Oncology, The Netherlands Cancer Institute, Amsterdam, 1066 CX, The Netherlands; Department of Medical Oncology, The Netherlands Cancer Institute, Amsterdam, 1066 CX, The Netherlands; Department of Pharmacy and Pharmacology, The Netherlands Cancer Institute, Amsterdam, 1066 CX, The Netherlands; Department of Pharmacology, Princess Máxima Center for Pediatric Oncology, Utrecht, 3584 CS, The Netherlands; Department of Clinical Pharmacy, University Medical Center Utrecht, Utrecht University, Utrecht, 3584 CX, The Netherlands; Department of Medical Oncology, The Netherlands Cancer Institute, Amsterdam, 1066 CX, The Netherlands; Department of Medical Oncology, University Medical Center Utrecht, Utrecht University, Utrecht, 3584 CX, The Netherlands

**Keywords:** oncology, early-phase clinical trials, clinical trial dissemination, discontinued compounds

## Abstract

**Introduction:**

Early-phase oncology clinical trials are often underrepresented in studies regarding publication of trial results. This study investigated the publication status of early-phase clinical trials, focusing on trials of compounds discontinued in clinical development.

**Methods:**

Two retrospective cohorts were formed: one included early-phase trials supporting European Medicines Agency marketing authorization applications (2017-2020), while the second cohort included early-phase trials of discontinued compounds. Trials were matched with database registry and their corresponding publications.

**Results:**

The first cohort consisted of 73 trials. Nine ongoing trials were excluded. No full publication or full report was found for 11% (*n* = 7/64) of trials. Median time from registered closure date to publication of trial results as full publication or full report was 109 days (IQR −733 to 597). Trials sponsored by large-size firms (HR 2.25 [95% CI, 1.23-4.1]), with >100 patients (HR 2.15 [95% CI, 1.19-3.88]) and duration >2 years (HR 6.83 [95% CI, 3.03-14.38]) were more likely to be published. The cohort with discontinued compounds consisted of 66 clinical trials. After exclusion of 9 ongoing trials, 39% (*n* = 22/57) lacked full publication or full report. Median time from registered closure date to publication of trial results as full publication or full report was 693 days (IQR 406–963). No covariates influencing publication status were found.

**Conclusions:**

Timely and complete publication of early-phase clinical trial results remains inadequate. Forty percent of trials with discontinued drugs are not publicly reported, and even among approved compounds, 11% of early-phase trial results cannot be found in the public domain.

Implications for practice Timely and complete dissemination of early-phase clinical trial results remains inadequate. Trials supporting European Medicines Agency marketing authorization applications had a median time of 109 days between registered closure to result dissemination, with 11% remaining unpublished. Moreover, trials involving discontinued compounds had a median dissemination time of 702 days, and 39% remained unpublished. These findings underscore the need to strengthen dissemination practices and enforce existing regulations. Failure to disseminate undermines transparency, promotes unnecessary duplication of research, may expose participants to ineffective or unsafe strategies, and wastes human and financial resources.

## Introduction

Dissemination of clinical trial results is essential in the drug development process. It has long been recognized that researchers have an ethical obligation to disseminate clinical trial results, as outlined in the Declaration of Helsinki (from April 1997 onward, currently described in 36th paragraph, version 2024).^1^ This ethical framework aims to ensure that patient data contribute to the general body of knowledge, prevent unnecessary patient burden, and avoid wasting financial and human resources. Disregarding this ethical principle can ultimately lead to unnecessary duplication of research and impair evidence-based decision making.

This ethical framework is further detailed in various regulations and guidelines. Since 2016, the European Commission Guideline mandates a report of the clinical trial to be published within one year after closure date of the trial.^2^ Similarly, the Food and Drug Administration’s (FDA) and Medicines and Healthcare products Regulatory Agency require a sponsor to publish a trial summary within one year after the closure date of trial.^3^^,^^4^ Multiple databases are available to report these results, such as Clinicaltrials.gov (FDA), the Clinical Trials Information System (European Medicines Agency [EMA]) or the World Health Organization International Clinical Trials Registry Platform (ICTRP).^5–7^ In addition, guidelines endorsed the description of dissemination policies in protocols.^8–10^

Dissemination in a peer-reviewed format remains the golden standard.^11^^,^^12^ However, 8%-39% of clinical trials in any stage of clinical development are not being disseminated at all.^13–22^ If peer-reviewed report of clinical trial results is not feasible, it is possible to report the clinical trial results in the public database registries. However, only 49.5%-77.5% of clinical trials in the European Union (EU) Clinical Trial Register and FDA’s Clinicaltrials.gov are compliant with their regulatory obligation to report the results in a clinical database within the predefined timelines.^23–28^ This might be an overestimation as these database summaries are known to be inconsistent or incomplete.^29^^,^^30^ For both the clinical trials published in peer-reviewed journals and clinical trials published in database registries, literature is inconsistent as outcomes depend on the selection of clinical trials (eg, clinical trials of EMA or FDA approved drugs and/or clinical trials registered in Clinicaltrials.gov), type of publication (eg, peer-reviewed publication and/or abstracts and/or database registries), and the selected time period allowed for dissemination (eg, database cutoff of 1, 2, or 5 year(s) after completion). However, in general, Phase I trials are underrepresented in these studies, or not included at all when studies focus specifically on pivotal, later-stage clinical trials.

The primary aim of this study was to investigate the time to publication of early-phase clinical trials, with a specific focus on trials of compounds that were discontinued in clinical development. Risk factors potentially associated with late publication were explored.

## Methods

Data suitable for answering this research question are not easily accessible. Because Phase 1 studies are not obligated to be registered on Clinicaltrials.gov, the publicly accessible information of Phase 1 studies will be incomplete.^31^ Therefore, this retrospective cohort study consisted of 2 cohorts. First cohort were early-phase clinical trials supporting EMA marketing authorization (2017-2020). We aimed for complete coverage of these clinical trials, as we expected all Phase I and Phase I/II within this cohort to have been registered. However, the majority of early-phase clinical trials involve compounds that will eventually be discontinued during clinical development, as the clinical success rate of Phase I oncology clinical trials is approximately 5%.^32–34^ To capture data on these trials as well, a second cohort was formed consisting of early-phase clinical trials of compounds that were discontinued in clinical development.

Only publicly available sources of clinical trial data were used. Therefore, ethical approval was not required.

Definitions for full publication and full report are as follows: full publication if they were original research reports in peer-reviewed journals and included the primary outcomes, full reports if all primary outcome results were available in a publically accessible database registry or in a report from the sponsor, but not peer-reviewed.

### Compound selection and data extraction

The first cohort included compounds receiving EMA marketing authorization for oncological indications between 2017 and 2020, identified through EMA annual reports.^35–38^ This timeframe allowed at least 5 years for the publication to be completed after EMA marketing authorization. Compounds were excluded if they were registered outside the timeframe or if their initial EMA marketing authorization documents were not publicly available.

The second cohort was created from Cortellis by Clarivate, which is an AI-powered life science intelligence platform that provides curated data and analytics for a compound lifecycle.^39^ Compounds were selected if they were included between 2012 and 2014, allowing at least 10 years of development of the compound and reflecting the same timeframe of study initiation of compounds that received EMA marketing authorization. Inclusion criteria were registration as discontinued in clinical development (cutoff: January 20, 2026) and designed for oncological indications. Compounds indicated for solely preventative or supportive care, or solely researched in the preclinical setting, were excluded. Discontinuation was verified by a report of the pharmaceutical company or in a clinical trial publication, or if no new report about the compound was initiated for at least 10 years. Extracted variables are listed in Appendix SI.

### Clinical trials selection and data extraction

For the first cohort, clinical trials were selected if they were listed in the “Clinical Aspects” section in the EMA initial marketing authorization public assessment report, were Phase I or Phase I/II clinical trials, treated human patients with an oncological indication and if the objective was to collect safety and/or efficacy data. Exclusion criteria were; a sole objective to study food-effect or drug–drug interactions, Phase II or Phase III clinical trials, solely healthy volunteers, solely pharmacokinetics, and/or retrospective database studies.

Clinical trials of discontinued compounds were selected by searching compound identifiers in clinicaltrial.gov, ICTRP database, or EU Clinical Trials Register. Clinical trials were included if they involved patients with an oncological indication, were Phase I or Phase I/II clinical trials, and the primary objective was to collect safety and/or efficacy data. Exclusion criteria were similar to the first cohort. Extracted variables are listed in Appendix SI.

### Publication selection and classification

A literature search was performed in MEDLINE and Google Scholar using clinical trial and/or compound identifiers. Only studies reported in English were included. The earlies publication containing primary outcomes was considered the dissemination date. Publications were excluded if they contained no safety and/or efficacy analyses (eg, pharmacokinetic report only), were case reports, were an integrated analysis with no clinical trial-specific information, or contained no new data.

Publications were matched to database registries and/or EMA public assessment reports using clinical trial identifiers. If no clinical trial identifier was found in the publication, the clinical trial was matched based on compound name, sample size, dosing schedule, number of arms, and primary and secondary outcomes.

Publications were hierarchically classified as follows: #1a full publication, #1b full report, #2 partial publication, #3 conference abstract, or #4 not reported. Publications were classified as full publication if they were original research reports in peer-reviewed journals and included the primary outcomes (#1a). Partial publications contained incomplete descriptions of the primary outcomes (#2) in a peer-reviewed journal. These may, for example, be analyses of only one included treatment arm. If all primary outcome results were available in the database registry or in a report from the sponsor, the trial was considered a full report (#1b). If multiple publications were found of the same trial, the highest ranked category was prioritized. For example, if the full trial results were first published as conference abstract, but thereafter published in a peer-reviewed journal, the trials publication was ranked as full publication (#1a). If a trial was published as full publication (#1a) and full report (#1b), the first date of dissemination was registered. Only if trials remained unmatched to any type of publication, they were listed as (#4) not reported. All abstracts and publications were screened by ML. Extracted variables are listed in Appendix SI.

### Statistical analysis

Descriptive statistics characterized the compounds and clinical trials, stratified by compound development outcome (EMA marketing authorization vs discontinued). Clinical trials that were registered as “active, not recruiting” were excluded. The primary endpoint was the dissemination status as full publication (#1a) or full report (#1b) within 1 year after registered closure date of the clinical trial. Secondary endpoints were dissemination within 2 and 5 years. Cox proportional Hazard models assessed time from registered closure to dissemination. Adjustments were included for clustering of clinical trials of one compound, if necessary. It should be noted that dissemination date of clinical trial results may precede the registered closure date of the trial, resulting in a negative value/number of days for the time from closure to dissemination. Covariates included; firm size, trial size, year of study initiation, study duration, and study status. Covariate selection and description are listed in Appendix SI. In the time-to-event analyses, clinical trials that did not report a dissemination date were censored at the time of analysis (January 20, 2026). All statistical analyses were performed with R version 4.5.1 (R Core Team, 2024). A 2-sided *P* < .05 was considered statistically significant.

## Results

This study included 2 cohorts that consisted of 73 Phase 1 trials of compounds that received EMA marketing authorization and 66 trials of compounds that were discontinued in clinical development (Figure 1). The full list of compounds can be found in Appendix SII, Tables S1 and S2, and the full list of compound- and clinical trial characteristics in Table 1.

**Figure 1.**
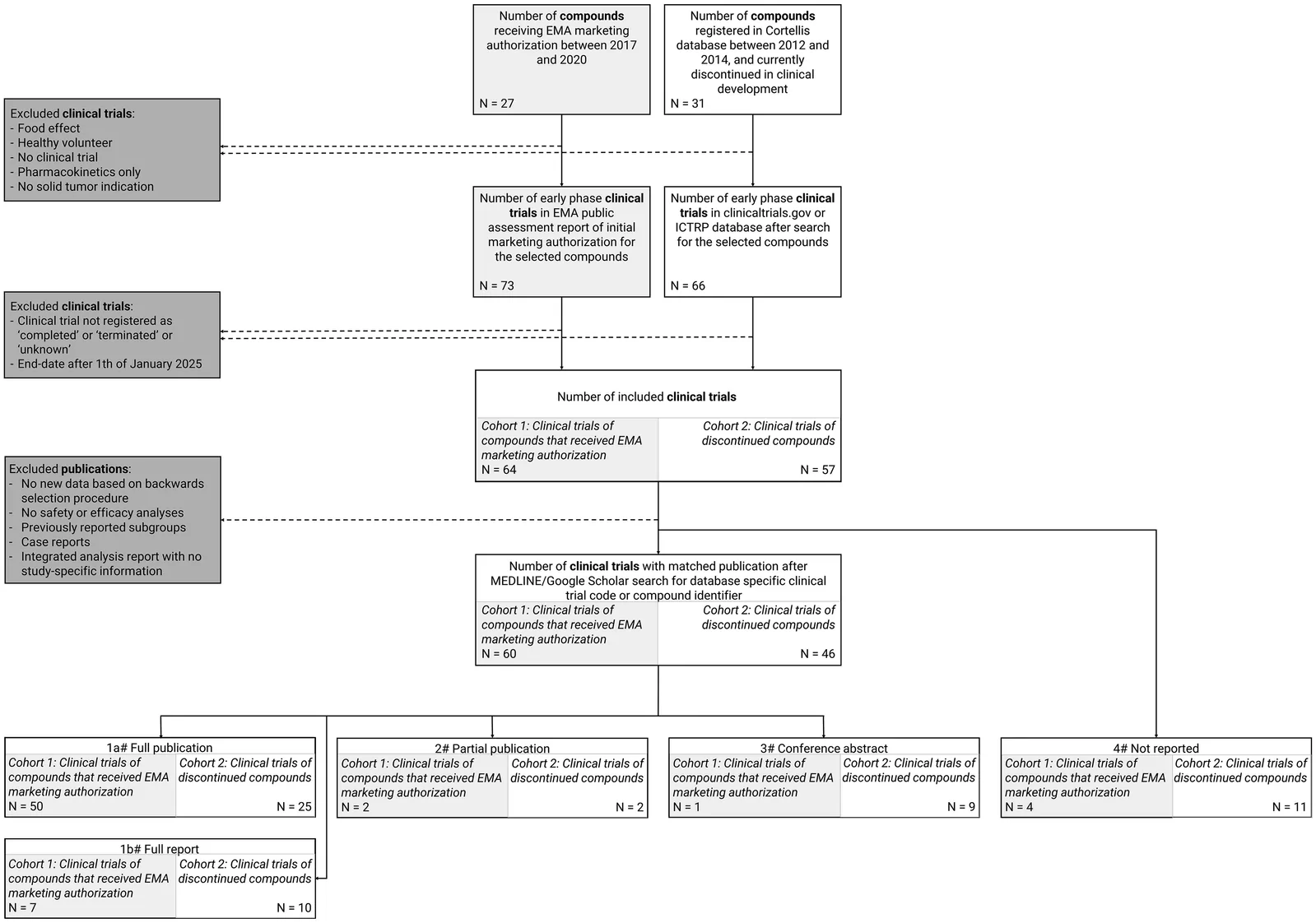
Flowchart of clinical trial selection and publication selection. EMA, European Medicines Agency; ICTRP, international clinical trials registry platform.

**Table 1.** Compound and clinical trial characteristics. Data are presented as number (percentage), unless stated otherwise.

	EMA marketing authorized	Discontinued in development
**Compounds characteristics**		
** Number of compounds**	27	31
** Orphan designation**	6 (22)	NA
** Therapy types**		
** Targeted therapy**		
** Small molecule**	14 (52)	11 (35)
** Other**	6 (22)	6 (19)
** Immunotherapy**		
** Immune checkpoint inhibitor**	4 (15)	0 (0)
** Monoclonal antibody**	0 (0)	2 (6)
** Bi-trispecific antibody**	0 (0)	5 (16)
** Antibody drug conjugate**	1 (4)	7 (23)
** Radiopharmaceutical**	1 (4)	0 (0)
** Photodynamic therapy**	1 (4)	0 (0)
** Company size**		
** Small**	7 (26)	6 (19)
** Large**	20 (74)	22 (71)
** Unknown**	0 (0)	3 (10)
**Clinical trial characteristics**		
** Number of included clinical trials**	64	57
** Number of clinical trials per compound [IQR]**	3 [2-4]	2 [2-3]
** Median time to publication of full publication, full report, partial publication or conference abstract after registered closure date of clinical trial in database registry (days) [IQR]**	−106 [−4230 to 2861]	702 [263–2200]
** Median duration of clinical trial (days) [IQR]**	1742 [851-2360]	955 [623-1371]
** Duration of clinical trial**		
** 0-2 years**	12 (19)	20 (36)
** 2-5 years**	24 (38)	31 (54)
** >5 years**	28 (44)	6 (11)
** Clinical trials that stopped development**		
** After Phase I drug development**	NA	21 (37)
** After Phase I/II drug development**	NA	13 (23)
** After Phase II drug development**	NA	21 (37)
** After Phase III drug development**	NA	2 (4)
** Clinical trial phase**		
** Phase I**	54 (84)	46 (81)
** Phase I/II**	10 (16)	11 (19)
** Trial size**		
**≤100 patients**	46 (72)	45 (79)
** >100 patients**	18 (28)	12 (21)
** Year of study start**		
** <2010**	16 (25)	0 (0)
** 2010-2015**	36 (56)	36 (63)
** >2015**	12 (19)	21 (37)
** Clinical trial status**		
** Completed**	60 (94)	36 (63)
** Terminated**	3 (5)	21 (37)
** Unknown**	1 (2)	0 (0)

Abbreviation: IQR, interquartile range.

### Trials of compounds that received EMA marketing authorization

#### Time to publication

Of the 73 clinical trials, 90% (*n* = 66/73) were registered in the database as “completed” (*n* = 60) or “terminated” (*n* = 3) or the study status was not registered in the database registry (*n* = 3). Seven trials were registered as “active, not recruiting” and were therefore excluded from these analyses. These 7 trials all had (part of) their results published; 4 clinical trials were published as full publication, 2 were published as partial publication and one had results published in a conference abstract. To account for at least one year of dissemination time, the included trials were filtered on registered closure date before January 1, 2025. This accounted for 64 included clinical trials.

A total of 11% (*n* = 7/64) of these trials were not disseminated as a full publication (#1a) or full report (#1b) before data cutoff (January 20, 2026). These clinical trials were not fully published or had no full report in the public domain: a partial publication (#2) was found of 2 clinical trials, one clinical trial was published as conference abstract (#3), and four were not reported (#4) at all. Median time between registered closure date and dissemination as full publication or full report was 109 days (IQR −733 to 587).

Within one year after registered closure date in the database registry, 42% (*n* = 27/64) of the early-phase registration trials of compounds that received EMA marketing authorization had not been disseminated as full publication (#1a) or full report (#1b). Two years after registered closure date of the clinical trial 31% (*n* = 20/64), and 5 years after registered closure date of the clinical trial 16% (*n* = 10/64) were not published (Figure 2).

**Figure 2.**
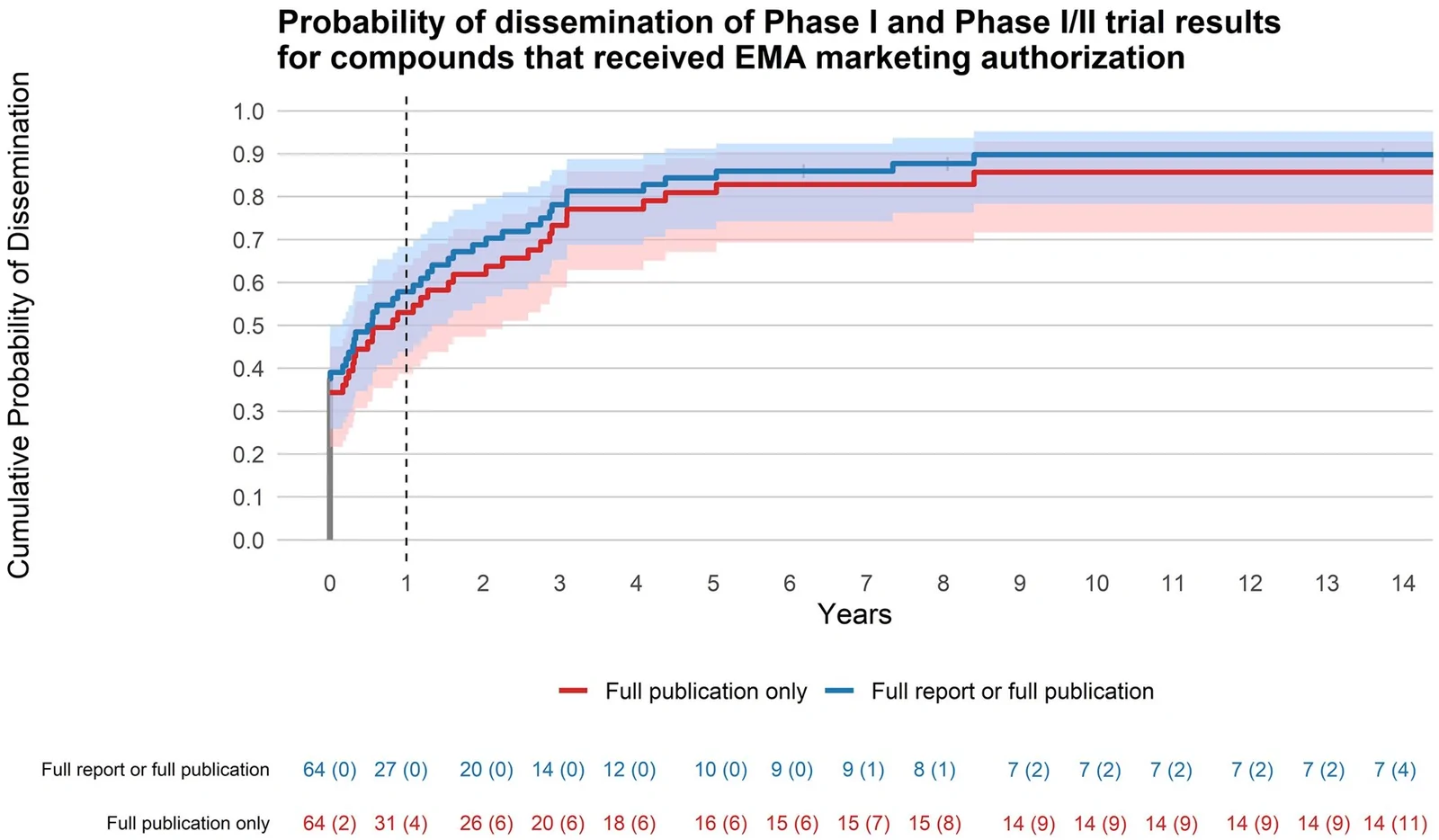
Time from registered closure date to dissemination date of compounds that received EMA marketing authorization. The cumulative probability of dissemination is shown as date of dissemination of the full publication or the full report as event in blue, and full publication only in red. Clinical trials that are not published were censored on January 20, 2026. Lighter colored area indicates the 95% CI. The vertical dashed line indicates one year after registered closure date of the clinical trial. The numbers at risk table shows the total numbers of trials that are not disseminated at each specific time point, including the number of censored trials which are displayed in brackets. Dissemination date of clinical trial results may precede the registered closure date of the trial, resulting in a negative value/number of days for the time from closure to dissemination.

#### In-depth analysis

An in-depth assessment highlights several observations. First, as depicted in Figure 2, 38% (*n* = 24/64) of the clinical trials were disseminated on or disseminated before the registered closure date of the clinical trial. In contrast, 5 studies (*n* = 5/64, 8%) were still not disseminated 10 years after registered closure date in the clinical database registry. Three of these trials were terminated due to slow accrual or for unknown reasons, with no data found in the public domain. These trials together included 40 patients that participated according to the database registry (NCT00971191, *n* = 22; NCT01110603, *n* = 12; NCT01227941, *n* = 6). The 2 remaining studies were registered as completed; one had a conference abstract with results of all 19 patients that entered the study according to the database registry^40^ (NCT01294735), and no results were found in the public domain for the other study (NCT00826878).

Secondly, four compounds (cemiplimab, lorlatinib, durvalumab, and avelumab) received EMA marketing authorization without any results of their early-phase clinical trials, which were used for this marketing authorization, disseminated in the public domain. Sponsors are not obliged to publish the results before receiving EMA marketing authorization.^2^ Interestingly, lorlatinib received EMA marketing authorization based on only a single Phase I/II clinical trial of 364 patients with ALK-positive or ROS1-positive non-small cell lung cancer (NCT01970865). This trial was disseminated after the EMA marketing authorization was granted (the date of publication: December 1, 2019, date of EMA marketing authorization: May 6, 2019). Signifying the incomplete dissemination, the cemiplimab Phase I clinical trial started in 2015 and ended in 2019. There were multiple partial publications (#2) and abstracts (#3) published between May 2016 and May 2021 with results of 196 patients in total.^41–44^ No full publication (#1a) or full report (#1b) followed until data cutoff (January 20, 2026), while there were 397 patients included in the EMA public assessment report and 398 patients in the database registry.

For early-phase clinical trials used for EMA marketing authorization, a significant slower dissemination of clinical trials of compounds owned by small-size firms was observed compared to clinical trials of compounds owned by larger size firms (HR 2.25 [95% CI, 1.23-4.1], *P* = .01). Clinical trials that included more than 100 patients were more likely to be disseminated as full publication (#1a) or full report (#1b) compared to clinical trials with less than 100 patients (HR 2.15 [95% CI, 1.19-3.88]). Early-phase clinical trials that started after 2016 were more likely to be disseminated compared to clinical trials initiated before 2010 (HR 2.54 [95% CI, 1.15-5.63]). Early-phase clinical trials with a duration time >5 years were more likely to get disseminated compared to trials that run for 0-2 years (HR 6.83 [95% CI, 3.03-15.38]). None of these studies were terminated. Correction for potential clustering within compounds showed similar hazards. Results are presented in Appendix SIII, Table S3. There was no difference in time between registered closure and dissemination for clinical trials that started between 2010 and 2015 vs before 2010 or after 2015.

### Trials of compounds that were discontinued in clinical development

#### Time to publication

The second cohort consisted of 66 clinical trials. In total, 86% (*n* = 57/66) clinical trials were registered in a database registry as “completed” (*n* = 36) or “terminated” (*n* = 21), and were completed before January 1, 2025. Five clinical trials were registered as “active, not recruiting” and were therefore excluded from the analyses. Of these, 2 had their results disseminated in a full publication, and 3 had part of their results published in a conference abstract.

In total, 39% (*n* = 22/57) of the trials were not disseminated as full publication (#1a) or full report (#1b). These clinical trials were not fully published or had no full report in the public domain: a partial publication (#2) was found of 2 clinical trials, only a conference abstract (#3) of 9 clinical trials, and 11 had no results disseminated (#4) in the public domain at all. Median time between registered closure and dissemination as full publication or full report was 693 days (IQR 406–963).

Within 1 year after registered closure date of clinical trial in the database registry, 77% (*n* = 44/57) had not been disseminated as full publication (#1a) or full report (#1b). After 2 years, 60% (*n* = 34/57) was not disseminated, and 40% (*n* = 23/57) after 5 years (Figure 3).

**Figure 3.**
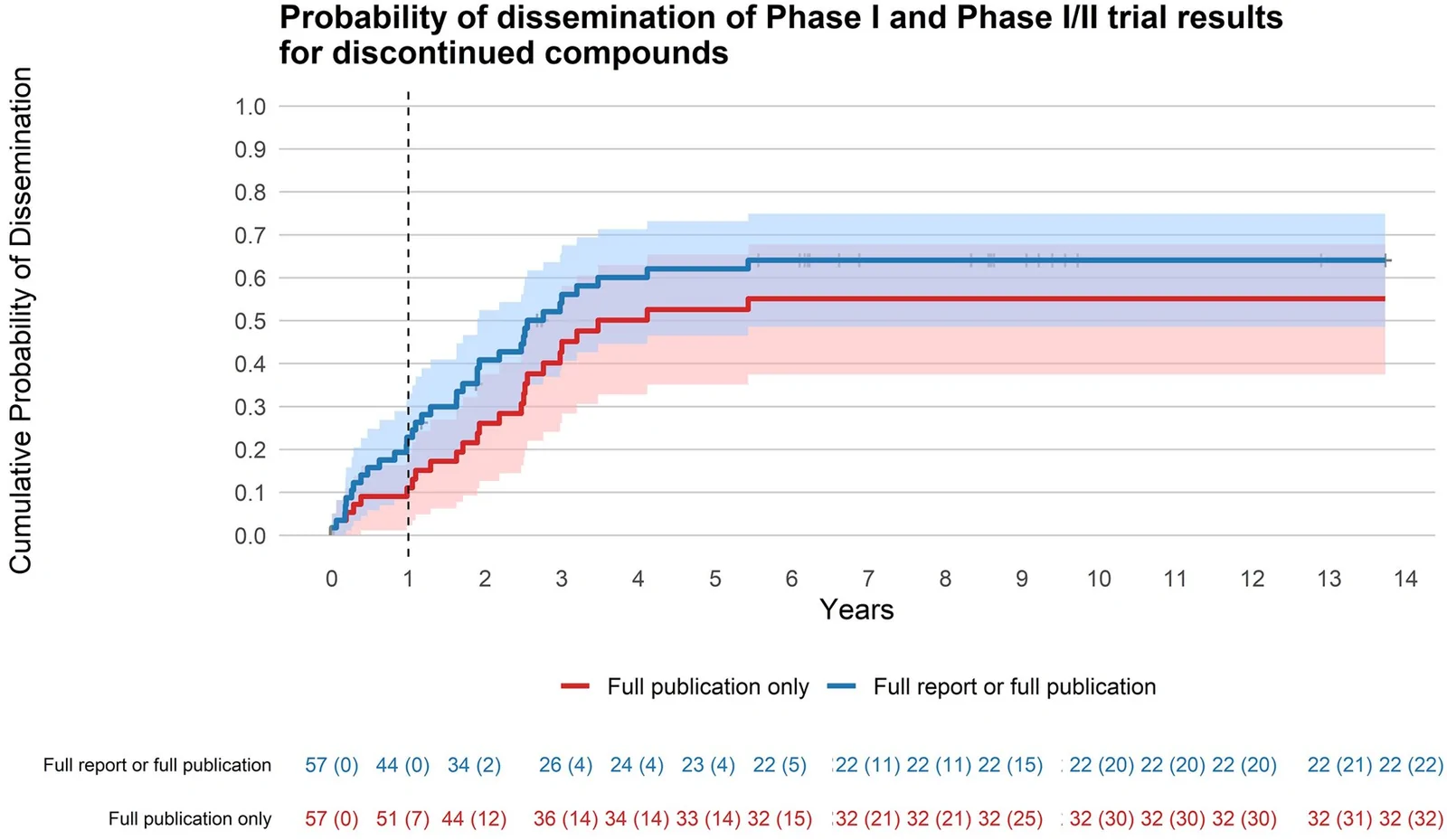
Time from registered closure date to dissemination date of compounds that were discontinued in clinical development. The cumulative probability of dissemination is shown with date of dissemination of the full publication or the full report as event in blue, and full publication only in red. Clinical trials that are not published were censored on January 20, 2026. Lighter colored area indicates the 95% CI. The vertical dashed line indicates 1 year after registered closure date of the clinical trial. The numbers at risk table shows the total numbers of trials that are not disseminated at each specific time point, including the number of censored trials which are displayed in brackets. Dissemination date of clinical trial results may precede the registered closure date of the trial, resulting in a negative value/number of days for the time from closure to dissemination.

#### In-depth analysis

An in-depth assessment did reveal minimal inconsistencies. Only one clinical trial was disseminated before registered closure date (*n* = 1/57, 2%). This trial was terminated in June 2020 (NCT01948297), but results were disseminated as full publication in May 2019. Additionally, 5 other studies only presented their results in a conference abstract before registered closure; four studies published the primary outcome data on (almost) all included patients in a conference abstract within 1 year before registered closure date (NCT02014909, NCT03415126, NCT01363024, NCT02304393), and one study only published data of 44 out of 610 included patients in a conference abstract 3 years before registered clinical trial closure.^45^ In contrast, one terminated clinical trial was completed in 2016, but the results of all 19 patients were presented in a conference abstract in 2021 (NCT02110563).^46^ The longest time between registered closure date and dissemination date was a Phase I study with 7 patients that ended in 2016, where the results were published in 2022.^47^

For early-phase clinical trials of compounds that were discontinued in clinical development, there was no significant difference in time to publication based on firm sizes, whether clinical drug development continued after Phase I, therapy type, clinical trial size, year of study start, duration of study, or study status. Correction for potential clustering within compounds showed similar hazards. Results are presented in Appendix SIII, Table S4.

## Discussion

These 2 retrospective cohorts showed that the timely and complete dissemination of early-phase clinical trials in oncology is still insufficient, raising ethical concerns. When patients enroll in early-phase clinical trials, they are exposed to experimental treatments, invasive procedures, such as research biopsies, and to potential toxicity. These patients participate motivated by contributing to the development of new compounds for future cancer patients and providing insights into drug response and resistance mechanisms. Failing to publish clinical trial results undermines the ethical justification for exposing patient to these risks and compromises the value of their contribution.^48–50^

Our first cohort included clinical trials of compounds that received EMA marketing authorization and concluded that dissemination of these trials was consistent with previous literature. Overall, 89% of clinical trials were disseminated, and the median time between registered closure date and dissemination as full publication or full report was 109 days (IQR −733 to 587). The majority of the clinical trials were disseminated within one year after study closure. However, in this cohort, inconsistencies in reporting of clinical trials in the database registries limit the interpretability of the results. For example, 38% of the clinical trials of compounds that received EMA marketing authorization were disseminated on or before the registered closure date in the database, which explained the negative time between registered closure and dissemination dates in this cohort. This may seem surprising, as the closure of a clinical trial is expected before its results are disseminated. However, the database registry’s recorded closure date marks the full closure of the clinical trial, while this is not necessarily the completion of primary (and/or secondary) outcome. As a result, data for the primary outcome can be analyzed and disseminated before the registered trial closure. The trial may remain open to gather additional follow-up data, such as long-term survival or toxicity outcomes. Another example of these inconsistencies in database registration, is the finding that 3 clinical trials were still registered as “active, not recruiting” while their primary results were published in a peer-reviewed publication. Requiring database registries to record the last patient inclusion or finalization of the primary endpoint could help improve transparency of clinical trial reporting.

Surprisingly, four compounds did not have their Phase I clinical trial disseminated as full publication or full report before they received EMA marketing authorization. This might be perceived as unwanted, because clinicians depend on the publicly available information when a new compound reaches marketing authorization. Guidelines are frequently revised at later stages after EMA marketing authorization, requiring clinicians to rely on published evidence or registered study results of new compounds in order to make a well-informed decision regarding their treatment plan for patients. The limited and/or late availability of information of a new compound in the public domain is therefore potentially harmful to the patient.

Comparable with previous reports, clinical trials of compounds that received EMA marketing authorization had higher risk of not having the results disseminated in the public domain if they had a small sample size.^51^ Reports differ on an improvement of time to publish clinical trial results, over time. A recent Cochrane Systematic Review by Showell et al. (2024) did not find an improvement of the time to publication (specified as “time from study completion” or “time from enrollment of first participant” to “publication in a journal and/or conference abstract”) over the last decades; however, they did find high heterogeneity across studies.^52^ Our study found that recent clinical trials of compounds that received EMA marketing authorization were more likely to be published than clinical trials that were initiated before 2010, whereas no such improvement was observed for clinical trials of discontinued compounds.

Our second cohort included early-phase clinical trials of discontinued compounds and concluded that only 61% of these clinical trials were fully disseminated. This is lower than reported in current literature on publication of all clinical trials across all study phases, where 77.5%-94.2% was reported to be published in a peer reviewed journal and/or full report.^13^^,^^14^^,^^16^^,^^27^^,^^51^ This is worrisome, as the vast majority of compounds entering Phase I (∼95%) will ultimately fails, making this low percentage of dissemination of Phase I results likely applicable to most early-phase clinical trials. A decision not to publish can ultimately lead to unnecessary duplication of research and impair evidence-based decision making.

Investigators might not disseminate the early-phase clinical trial results due to insufficient resources, inadequate collaboration, limited motivation and/or practicalities.^53–55^ This high number of unpublished early-phase clinical trials cannot be attributed solely to potential negative outcomes of these clinical trials, as progression beyond Phase I was not associated with time to publication in our analysis. Notably, 48% of clinical trials of the compounds were discontinued in development after Phase I in our cohort, which is comparable with literature.^34^^,^^56^ Thus, discontinuation of compounds may be a key factor influencing the dissemination of clinical results, despite being infrequently considered in previous studies. Instead, most studies focus primarily on the primary outcome (positive vs negative) results.

This study should be interpreted in light of some limitations. Heterogeneity in the 2 cohorts introduced potential bias preventing direct comparison. Therefore, an in-depth assessment of each cohort was performed. Studies were selected if the trial ended at least 1 year before the data cutoff. However, as the median time to publication of the second cohort exceeded 1 year, this might have influenced the results. For the compounds that received EMA marketing authorization, no censoring was observed until 7 years after registered closure date, suggesting minimal influence. However, multiple discontinued compound studies needed censoring, as they were not published at time of analysis (*n* = 22/57). This censoring reflects the number of unpublished or incompletely published studies. Only 2 trials were censored within 2 years after registered closure date and might have been published if the data cutoff allowed more time to publish. Finally, the publication search was performed in MEDLINE and Google Scholar, which may result in some meeting abstracts are not found, specifically from small conferences.

To conclude, timely and complete dissemination of early-phase clinical trials in oncology is insufficient. Particularly, results of clinical trials involving compounds that were discontinued in clinical development were underreported, even though they represent the majority of compounds tested in Phase I. The dissemination of early-phase clinical trials of compounds that received EMA marketing authorization seemed consistent with previous reports, as only 11% was not published as full publication or full report, and most were published within the first year after end of study. However, the interpretability of these results is limited by inconsistencies in the reporting of clinical trials in database registries. Introducing new variables into registration databases, such as “inclusion date of last patient” or “interim analysis date of primary outcome,” might improve the consistency of reporting on time to publication. Additionally, improving the time to publication of early-phase clinical trials requires reducing the barriers encountered during the publication process. Previously, it was highlighted that there is need for improved clarity on the responsibility of disseminating clinical trial results.^54^ Especially in situations where compounds are discontinued and/or industry partners withdraw due to financial constraints or insolvency, oncologists often find it unclear whether the sponsor, lead principal investigator or individual sub-investigator bear responsibility for publication. Dedicated journal sections, such as this journal’s *Clinical Trial Results* section, can help facilitate dissemination in such cases. Clear guidance may improve dissemination practices, especially for early-phase clinical trials. Future initiatives could aim to identify feasible and impactful strategies to improve the publication of early-phase clinical trial results, thereby enhancing transparency in oncology.

## Supplementary Material

oyag361_Supplementary_Data

## Data Availability

All data analyzed in this study were obtained from MEDLINE database, the Clinicaltrials.gov database and Google Scholar.
