# Supplementary material for: The insufficient publication of early-phase clinical trials in oncology
Source: Oncologist. 2026 Sep 12;31(10):oyag361. doi: 10.1093/oncolo/oyag361 (PMC13623515; doi:10.1093/oncolo/oyag361)
Supplement: Supplementary file 1 [file oyag361_supplementary_data.zip › Appendices.pdf]

The insufficient publication of early-phase clinical trials in oncology

Running title: Insufficient publication of clinical trials in oncology

Authors: Merel J.J. Lucassen[1], Tim Schutte[1], Alwin D.R. Huitema[2,3,4], Neeltje Steeghs[1,5]

Affiliations

1. Department of Medical Oncology, The Netherlands Cancer Institute, Amsterdam, The Netherlands.
2. Department of Pharmacy & Pharmacology, The Netherlands Cancer Institute, Amsterdam, The Netherlands.
3. Princess Máxima Center for Pediatric Oncology, Utrecht, The Netherlands.
4. Department of Clinical Pharmacy, University Medical Center Utrecht, Utrecht University, Utrecht, The Netherlands.
5. Department of Medical Oncology, University Medical Center Utrecht, Utrecht University, Utrecht, The Netherlands.

Journal: The Oncologist

Appendix I

Data extraction from compounds selection documents

The following variables were extracted from the initial EMA marketing authorization documents for the first cohort; compound identifiers, therapy type, therapy route, investigational medicinal product (IMP) owner, Committee for Medicinal Products for Human Use (CHMP) application date, EMA marketing authorization granted date and European Commission (EC) approval date.

For the second cohort, all available names and/or codes for the compound were extracted from the discontinuation reports or publications. These included the current IMP owner's name for the compound, the past IMP owners' names, the generic name and/or developmental code(s).

Data extraction from clinical trials registry

In the first cohort, the clinical trial number referenced in the EMA public assessment report was matched to the Clinicaltrials.gov or other database identifier, when available. For the second cohort, clinical trials were selected based on compound search in the Clinicaltrials.gov and/or ICTRP registry. The following clinical trial specific variables were extracted from the database registry for both cohorts; clinical trial sponsor, therapy type, therapy route, clinical trial phase, clinical trial identifiers, clinical trial status (e.g. completed, terminated, recruiting), start-date clinical trial, closure date of the clinical trial (actual, or ongoing clinical trials were censored on January 20th 2026), full results posted (yes/no), date of results posted.

Data extraction from publications

For both cohorts, the following variables were extracted from each publication; the type of publication, data cut-off, date of last inclusion and date of publication.

Statistical analysis; covariates

Covariates included; firm size (annual revenue >1000M dollar (large) versus <1000M dollar (small) at start of clinical trial in report of pharmaceutical company), trial size (>100 patients versus <100 patients), year of study initiation (before 2010, 2010-2015 or after 2015), study duration (0-2 years, 2-5 years or more than 5 years) and study status (ongoing, completed or terminated). These covariates were selected based on their correlation to the time between registered closure date and dissemination, as reported in previous studies. [1-4] Only the covariate 'study duration' was not reported as covariate in previous analyses. This covariate was included as we hypothesized that a longer study duration could indicate more chance of not publishing the study.

1 Omae K, Kataoka Y, Tsujimoto Y, et al. Publication statuses of clinical trials supporting fda-approved immune checkpoint inhibitors: A meta-epidemiological investigation. BMC Cancer 2019;19:998.
2 Showell MG, Cole S, Clarke MJ, et al. Time to publication for results of clinical trials. Cochrane Database Syst Rev 2024;11:Mr000011.
3 Huang RQ, Zhou Y, Zheng HX, et al. Transparency of clinical trials in pancreatic cancer: An analysis of availability of trial results from the clinicaltrials.Gov database. Front Oncol 2022;12:1026268.
4 Dwan K, Gamble C, Williamson PR, Kirkham JJ. Systematic review of the empirical evidence of study publication bias and outcome reporting bias - an updated review. PLoS One 2013;8:e66844.

Appendix II

Supplementary Table 1. List of included compounds that received EMA marketing authorization

Generic Name	Therapy type	Therapy route	Firm IMP holder	Firm size	Number of trials
Avapritinib	Small molecule	Oral	Blueprint Medicines	Small	3
Trastuzumab Deruxtecan	Antibody Drug Conjugate	Intravenous	Daiichi Sankyo	Large	4
Darolutamide	Targeted therapy – Other	Oral	Bayer	Large	3
Pertuzumab/ trastuzumab	Targeted therapy – Other	Subcutaneous	F. Hoffman – La Roche AG	Large	1
Selpercatinib	Targeted therapy – Other	Oral	Lilly	Large	1
Entrectinib	Small molecule	Oral	F. Hoffman – La Roche AG	Large	3
Tucatinib	Small molecule	Oral	Seagen	Large	3
Cemiplimab	Immune checkpoint inhibitor	Intravenous	Regeneron	Large	1
Lorlatinib	Small molecule	Oral	Pfizer	Large	1
Talazoparib	Targeted therapy – Other	Oral	Pfizer	Large	5
Dacomitinib	Small molecule	Oral	Pfizer	Large	5
Larotrectinib	Small molecule	Oral	Bayer	Large	2
Brigatinib	Small molecule	Oral	Takeda Pharmaceuticals	Large	1
Durvalumab	Immune checkpoint inhibitor	Intravenous	AstraZeneca	Large	1
Encorafenib	Small molecule	Oral	Pierre Fabre	Large	3
Apalutamide	Targeted therapy – Other	Oral	Johnson & Johnson	Large	4
Binimetinib	Small molecule	Oral	AstraZeneca	Large	3
Neratinib	Small molecule	Oral	Puma Biotechnology	Small	4
Rucaparib	Targeted therapy – Other	Oral	Clovis Oncology UK limited	Small	4
Abemaciclib	Small molecule	Oral	Lilly	Large	4
Avelumab	Immune checkpoint inhibitor	Intravenous	Merck Group	Large	2
Tivozanib	Small molecule	Oral	EUSA Pharma	Small	4

Ribociclib	Small molecule	Oral	Novartis	Large	3
Lutetium Oxodotreotide	Radiopharmaceutical	Intravenous	Advanced Accelerator Applications	Small	1
Atezolizumab	Immune checkpoint inhibitor	Intravenous	F. Hoffman – La Roche AG	Large	1
Padeliporfin	Photodynamic therapy	Intravenous	Steba Biotech	Small	1
Niraparib	Targeted therapy – Other	Oral	Tesaro UK Limited	Small	5

Supplementary Table 2. List of included discontinued compounds

Generic Name	Therapy type	Therapy route	Firm IMP holder	Firm size	Number of trials
ASP-9853	Small molecule	Oral	Astellas	Large	1
SAR-260301	Small molecule	Oral	Sanofi	Large	1
Cergutuzumab amunaleukin	Bi-trispecific antibody	Intravenous	F. Hoffman – La Roche AG	Large	2
Onvatilimab	Bi-trispecific antibody	Intravenous	Curis	Small	2
Zoligratinib	Small molecule	Oral	Debiopharm	Unknown	3
CDX-3379	Bi-trispecific antibody	Intravenous	Celldex Therapeutics	Small	5
GSK-3174998	Bi-trispecific antibody	Intravenous	Glaxosmithkline	Large	2
DCR-M1711	Targeted therapy – Other	Intravenous	Dicerna Pharmaceutical	Small	3
OPB-111077	Small molecule	Oral	Otsuka Pharmaceuticals	Large	7
Ravoxertinib	Small molecule	Oral	Genentech	Large	2
STM-434	Targeted therapy – Other	Intravenous	Santa Maria Biotherapeutics	Small	1
CC-90003	Small molecule	Oral	Celgene	Unknown	1
PF-06650808	Antibody Drug Conjugate	Intravenous	Pfizer	Large	1
LY-3164530	Bi-trispecific antibody	Intravenous	Lilly	Large	1
AZD-8835	Small molecule	Oral	AstraZeneca	Large	1
PF-06647263	Antibody Drug Conjugate	Intravenous	Pfizer	Large	1

Naquotinib	Small molecule	Oral	Astellas	Large	5
Tusamitamab ravtansine	Antibody Drug Conjugate	Intravenous	Sanofi	Large	8
Medi-4276	Antibody Drug Conjugate	Intravenous	AstraZeneca	Large	1
ERAS-007	Small molecule	Oral	Erasca	Unknown	4
Pogalizumab	Targeted therapy – Other	Intravenous	Genentech	Large	2
Cofetuzumab pelidotin	Antibody Drug Conjugate	Intravenous	AbbVie	Large	2
Vofatamab	Targeted therapy – Other	Intravenous	Genentech	Large	5
Enapotamab vedotin	Antibody Drug Conjugate	Intravenous	Genmab	Large	1
Selicrelumab	Targeted therapy – Other	Intravenous	F. Hoffman – La Roche AG	Large	3
Lirametostat	Small molecule	Oral	Constellation Pharmaceuticals	Small	2
PTX-35	Monoclonal antibody	Intravenous	Pelican Therapeutics	Small	1
DS-5573	Monoclonal antibody	Intravenous	Daiichi Sankyo	Large	1
DS-8273	Targeted therapy – Other	Intravenous	Daiichi Sankyo	Large	2
PF-06664178	Antibody Drug Conjugate	Intravenous	Pfizer	Large	1

Appendix III

Supplementary Table 3. Time to dissemination of Phase 1 clinical trials of compounds that received EMA marketing authorization

**Covariate**		Hazard Ratio	95% Confidence Interval
Firm size (Large versus small)		2.25	1.23–4.1*
Trial size (>100 patients versus ≤ 100 patients)		2.15	1.19–3.88*
Year of study start	<2010 versus >2015	2.54	1.15–5.63*
	Between 2010-2015 versus > 2015	2.03	1.0–4.14
	Between 2010-2015 versus < 2010	0.8	0.43–1.49
Run time study	<2 years versus 2-5 years	1.42	0.65–3.14
	2-5 years versus ≥5 years	4.8	2.56–9.02*
	<2 years versus ≥5 years	6.83	3.03–15.38*

Unadjusted Cox proportional Hazards model that included solely the covariates. Correction for potential clustering of clinical trials of the same compound did not influence any analysis.

Supplementary Table 4. Time to dissemination of Phase 1 clinical trials of discontinued compounds

**Covariate**		Hazard Ratio	95% Confidence Interval
Firm size (Large versus small)		1.29	0.53–2.96
Trial size (>100 patients versus ≤ 100 patients)		1.39	0.63–3.06
Year of study start	Between 2010-2015 versus > 2015	1.02	0.51–2.06
Runtime study	<2 years versus 2-5 years	1.35	0.65–2.83
	2-5 years versus ≥5 years	1.49	0.6–3.68
	<2 years versus ≥ 5 years	2.02	0.63–6.42
Study completed versus terminated		1.64	0.83–3.25
Development until Phase I versus until Phase II or Phase III		1.25	0.64–2.24

Unadjusted Cox proportional Hazards model that included solely the covariates. Correction for potential clustering of clinical trials of the same compound did not influence any analysis.
